# Identification of IFITM1 and IFITM3 in Goose: Gene Structure, Expression Patterns, and Immune Reponses against Tembusu Virus Infection

**DOI:** 10.1155/2017/5149062

**Published:** 2017-03-13

**Authors:** Anqi Wang, Lipei Sun, Mingshu Wang, Renyong Jia, Dekang Zhu, Mafeng Liu, Kunfeng Sun, Qiao Yang, Ying Wu, Xiaoyue Chen, Anchun Cheng, Shun Chen

**Affiliations:** ^1^Institute of Preventive Veterinary Medicine, Sichuan Agricultural University, Chengdu, Sichuan 611130, China; ^2^Avian Disease Research Center, College of Veterinary Medicine, Sichuan Agricultural University, Chengdu, Sichuan 611130, China; ^3^Key Laboratory of Animal Disease and Human Health of Sichuan Province, Sichuan Agricultural University, Chengdu, Sichuan 611130, China

## Abstract

As interferon-stimulated genes (ISGs), interferon-inducible transmembrane proteins 1 and 3 (IFITM1 and IFITM3) can effectively inhibit the replication of multiple viruses. Here, goose IFITM1 and IFITM3 were cloned and identified for the first time. The two proteins share the same topological structure and several important sites critical for the antiviral functions in other species are conserved in the goose. Goose IFITM1 and IFITM3 are most closely related to their respective orthologs in ducks; these proteins exhibited high mRNA transcript levels in immune-related tissues, including the thymus, bursa of Fabricius, and Harderian gland, compared to other tissues. Moreover, goose IFITM1 was highly constitutively expressed in gastrointestinal tract tissues, while goose IFITM3 was expressed in respiratory organs. Furthermore, goose IFITM3 was activated in goose peripheral blood mononuclear cells (PBMCs) infected with Tembusu virus (TMUV) or treated with Toll-like receptors (TLRs) agonists, while only the R848 and Poly (I:C) agonists induced significant upregulation of goose IFITM1. Furthermore, goose IFITM1 and IFITM3 were upregulated in the sampled tissues, to some extent, after TMUV infection. Notably, significant upregulation of goose IFITM1 and IFITM3 was detected in the cecum and cecal tonsil, where TMUV was primarily distributed. These data provide new insights into the immune effectors in geese and promote our understanding of the role of IFITM1 and IFITM3 in the defense against TMUV.

## 1. Introduction

Types I and II interferons (IFNs) are critical for establishing an antiviral state, which is mediated by downstream IFN-stimulated genes (ISGs) [1]. Viruses have evolved diverse strategies to escape immune defenses [2]. However, virus evasion of the interferon-inducible transmembrane proteins (IFITMs) restriction is not apparent.

Recently, IFITMs have become a popular research topic with the discovery that the immune-related IFITMs (IFITM1, IFITM2, and IFITM3) inhibit the early replication of multiple viruses, including influenza A virus (IAV), dengue virus (DENV), West Nile virus (WNV), severe acute respiratory syndrome coronavirus (SARS CoV), hepatitis C virus (HCV), vesicular stomatitis virus (VSV), Ebola virus (EBOV), and human immunodeficiency virus type 1 (HIV-1) [3–8]. IFITMs are a subfamily of a larger transmembrane protein family called Dispanins [9], which are generally considered to be comprised of IFITM1, IFITM2, IFITM3, IFITM5, and IFITM10 [10]. IFITM4P, a pseudogene, is located on mouse chromosome as IFITM6 and IFITM7 [11]. Moreover, IFITM1, IFITM2, and IFITM3, as viral restriction factors, are known as immune-related IFITMs, the antiviral activity of which is conserved from prokaryotes to vertebrates. In addition, the immune-related IFITMs exhibit high constitutive expression in target cells and are strongly induced by types I and II IFNs [12]. IFITMs have common domains that consist of N- and C-termini, two transmembrane domains, and a conserved cytoplasmic domain and share common properties: (1) the proteins contain a CD225 domain composed of transmembrane domain 1 (TM1) and a cytoplasmic domain and (2) two exons encode transmembrane polypeptides [13]. Among these domains, the N-terminal region plays an important role in the correct cellular localization of IFITM3. Removing the N-terminal 21 amino acids of IFITM3 results in a loss of association with the endosomal compartments and relocation to the cell surface, thereby abrogating its antiviral function [14]. IFITM1 possesses a shorter N-terminal region and predominately localizes at the cell periphery and in early endosomes [4]. The different cellular localization of IFITM1 and IFITM3 likely underlies their diverse antiviral efficiency against various viruses [15]. IFITM1 exerts more resistance to viruses that fuse at the plasma membrane or early endosome, such as HIV-1 [8]. However, IFITM3 is more proficient than IFITM1 at preventing infection by the late endosomal- or lysosomal-entering viruses, including IAV and DENV [3]. At present, IFITMs have been widely identified in vertebrates, and homologs of IFITMs are even found in bacteria [16]. However, little attention has been paid to IFITMs in birds. Only a small number of bird IFITM sequences have been deposited in public databases, including* Gallus gallus* IFITM1-like (GenBank: XM_001233949),* Anas platyrhynchos* IFITM1 (GenBank: KF584226),* Gallus gallus* IFITM3-like (GenBank: XM_420925),* Anas platyrhynchos* IFITM3 (GenBank: KF584228),* Serinus canaria* IFITM3 (GenBank: XM_009102512), and* Nipponia nippon* IFITM3 (GenBank: XM_009463652). In addition, it was reported that chicken IFITM3 [17] and duck IFITM3 [18] could restrict influenza viruses, but information regarding goose IFITMs was unavailable.

Tembusu virus (TMUV) is a newly emerging pathogenic virus that, together with DENV, WNV, Japanese encephalitis virus (JEV), and yellow fever virus (YFV), belongs to the genus* Flavivirus* [19]. The TMUV genome is a single-stranded RNA with an open reading frame that encodes three structural proteins, namely, the core (C), membrane (prM), and envelope (E) proteins, and seven nonstructural proteins [20]. In China, the virus was initially isolated from ducks and can cause severe egg drop syndrome in laying ducks, resulting in huge economic losses. However, recent studies showed that chickens, geese, and house sparrows are also susceptible to this virus [21–23]. Most importantly, serum samples from workers in the duck industry contained TMUV antibodies, which may indicate potential zoonotic transmission [24]. In view of the significant antiviral activity of IFITMs in the IFN-mediated innate antiviral defense and the lack of studies focusing on avian IFITMs, goose IFITM1 and IFITM3 were cloned for the first time. In addition, the amino acid sequences and topological structures of these proteins were predicted. To understand the evolutionary relationship of goose IFITM1 and IFITM3, a phylogenetic tree was constructed. Moreover, the tissue distribution of goose IFITM1 and IFITM3 in healthy geese was identified. It has been demonstrated that IFITMs play an important role in blocking the entry of pathogenic flaviviruses such as DENV and WNV. As TMUV is a novel member of* Flavivirus*, the interaction between TMUV and IFITM-mediated immune responses is unknown. Interestingly, we observed that goose IFITM1 and IFITM3, to different degrees, positively responded to TMUV infection both in vivo and in vitro. Collectively, our results promote the research of avian IFITMs and may provide the molecular foundation for further research regarding the contribution of goose IFITM1 and IFITM3 to the innate immune responses to TMUV infection.

## 2. Materials and Methods

### 2.1. Animals, Viruses, and Agonists

All geese were purchased from the waterfowl breeding center of Sichuan Agriculture University and provided with sufficient water, fodder, and vegetables prior to and during experiments. All experiments were approved by the Institutional Animal Care and Use Committee of Sichuan Agriculture University (Number XF2014-18), Sichuan, China.

TMUV was a kind gift of the Avian Diseases Research Center of Sichuan Agricultural University. The agonists R848 (InvivoGen, USA), Poly (I:C) (Sigma, USA), ODN2006 (InvivoGen, USA), and LPS (InvivoGen, USA) were used to mimic treatment with single-stranded RNA, double-stranded RNA, synthetic oligonucleotides, and lipopolysaccharides, respectively.

### 2.2. RNA Isolation and cDNA Synthesis

The animals were euthanized, and then the various tissues were rapidly removed and frozen in liquid nitrogen. Total RNA was extracted from tissues using TRIzol (Invitrogen, Carlsbad, CA, USA) following the manufacturer's protocol and then reverse transcribed using 5x All-in-One RT MasterMix (Abm, Canada), which could remove the genomic DNA. Finally, the synthetic cDNA was stored at −80°C until needed for cloning and quantitative real-time PCR (qRT-PCR).

### 2.3. Cloning of the Goose IFITM1 and IFITM3 Complete Coding Sequences (CDs)

The primers IFITM1-F, IFITM1-R, IFITM3-F, and IFITM3-R (all sequences of the optimal primers used in this study are listed in Table 1) were designed based on the conserved regions of their respective counterparts in chicken and duck to amplify the partial sequences of goose IFITM1 and IFITM3. Subsequently, the full-length cDNA of IFITM3 and the 5′-untranslated region (UTR) of IFITM1 were obtained by rapid amplification of cDNA ends (RACE). Briefly, the gene-specific primers (GSPs), including 5′GSP0, 5′GSP1, 5′GSP2, 3′GSP1, and 3′GSP2, were designed based on the obtained partial sequences. For 5′-RACE, a homopolymeric tail was added to the 5′-end of the synthetic cDNA using terminal deoxynucleotidyl transferase and dCTP (TaKaRa, Japan), and then the first-strand cDNA was synthesized using the primer 5′GSP0 and a HiScript 1st Strand cDNA Synthesis Kit (Vazyme, USA). The 5′-end of the target gene was obtained following two rounds of nested PCR using the primer 5′GSP1 with the Abridged Anchor Primer (AAP) and 5′GSP2 with the Abridged Universal Amplification Primer (AUAP). For 3′-RACE, the Adapter Primer (AP) was used to amplify the first-strand cDNA. The 3′-end of IFITM3 was also amplified by nested PCR with the primer pairs 3′GSP1/AP1 and 3′GSP2/AP2. The primer IFITM3-3′R, which was used for amplifying the 3′-UTR of IFITM3, was designed according to the predicted IFITM3 sequence from goose transcriptome data [25]. Finally, the complete CDs of IFITM1 and IFITM3 were amplified using Primer STAR Max DNA polymerase (TaKaRa, Japan) and were cloned into the pMD19-T vector (TaKaRa, Japan) for sequencing.

### 2.4. The Expression Profiles of Goose IFITM1 and IFITM3 in Healthy Geese

To evaluate the tissue expression profiles of IFITM1 and IFITM3, various tissues, including the brain, bursa of Fabricius, cecum, cecal tonsil, gizzard, heart, Harderian gland, kidney, liver, lung, muscle, pancreas, proventriculus, small intestine, skin, spleen, thymus, and trachea, were collected from two-week-old gosling and adult goose. The mRNA expression levels of IFITM1 and IFITM3 in the various tissues were detected by the Bio-Rad CFX96 Real-Time PCR Detection System (Bio-Rad, USA) and EvaGreen 2x qPCR MasterMix-No Dye Kit (Abm, Canada) according to the manufacturer's protocol. The relative expression of target genes was calculated using the Livak and Schmittgen 2^−ΔΔCT^ method [26] and was normalized to goose GAPDH.

### 2.5. The Effects of TMUV Infection and Agonist Treatment on the Expression of Goose IFITMs in Peripheral Blood Mononuclear Cells (PBMCs)

PBMCs were separated from goose blood using a goose Peripheral Blood Lymphocyte Separation Kit (TBD Sciences, China) according to the manufacturer's protocol. Then, PBMCs were cultured and maintained in RPMI 1640 medium (Gibco, USA) at 37°C in 5% CO_2_ overnight. Subsequently, the cells were treated with different Toll-like receptors (TLRs) agonists, including R848 (5 *μ*g/mL), Poly (I:C) (30 *μ*g/mL), ODN2006 (50 *μ*g/mL), and LPS (25 *μ*g/mL), or challenged with 50 *μ*L TMUV. Cells treated with PBS were used as an experimental control, and the treated cells were challenged with each agonist or virus in quadruplicate and incubated for 6 h. Finally, the cells from each treatment group were harvested for qRT-PCR analysis of the expression levels of goose IFITM1 and IFITM3.

### 2.6. The Effects of TMUV Infection on the Expression of Goose IFITMs In Vivo

Ten three-day-old goslings were divided into two groups with five goslings in each group. One group of goslings was infected with 500 *μ*L of TMUV (6.3 × 10^−6^ TCID_50_/mL), and the second group was inoculated with an equivalent amount of normal saline. Five days after infection (dpi), three goslings from each group were euthanized, and eight tissues (bursa of Fabricius, cecum, cecal tonsil, Harderian gland, lung, small intestine, spleen, and thymus) were collected immediately for RNA isolation and cDNA synthesis. The relative expression levels of IFITM1 and IFITM3 in each sample were assessed by qRT-PCR as described above.

### 2.7. Detection of TMUV Copy Number in TMUV-Infected Goslings

The full-length E gene from TMUV was amplified using Primer STAR Max DNA polymerase (TaKaRa, Japan) and subcloned into the pET-32a(+) vector. The temperature and primers were optimized with two negative controls (DEPC water) and two positive controls (GPV and H9N2 templates). Three separate dilution series (10^−1^ to 10^−8^) of recombinant plasmid were prepared for the establishment of standard curves. The viral copy numbers (log_10_) were normalized per 1 *μ*g of total RNA [27]. The viral copy number in TMUV-infected gosling tissues was quantified by qRT-PCR.

### 2.8. Bioinformatic Analysis

The potential open reading frames (ORFs) were predicted by ORF finder (https://www.ncbi.nlm.nih.gov/gorf/gorf.html), and the corresponding amino acid sequences of cDNA were translated with DNAMAN software. The molecular weight was calculated using ExPASy (http://www.expasy.org/tools/). The transmembrane helices prediction was performed by TMHMM 2.0 (http://www.cbs.dtu.dk/services/TMHMM/). The nucleotide sequences of IFITM1 and IFITM3 from numerous species were compared by pairwise identity analysis using the Species Demarcation Tool [28]. The multiple alignment analysis of amino acid sequences was performed using ClustalW [29]. The phylogenetic evolutionary tree was constructed by MEGA6 using the neighbor-joining method [30]. The qRT-PCR data were analyzed by Bio-Rad CFX Manager Software and GraphPad Prism 5 with an unpaired two-tailed *t*-test.

## 3. Results

### 3.1. Molecular Cloning and Identification of IFITM1 and IFITM3

**Figure 1:**
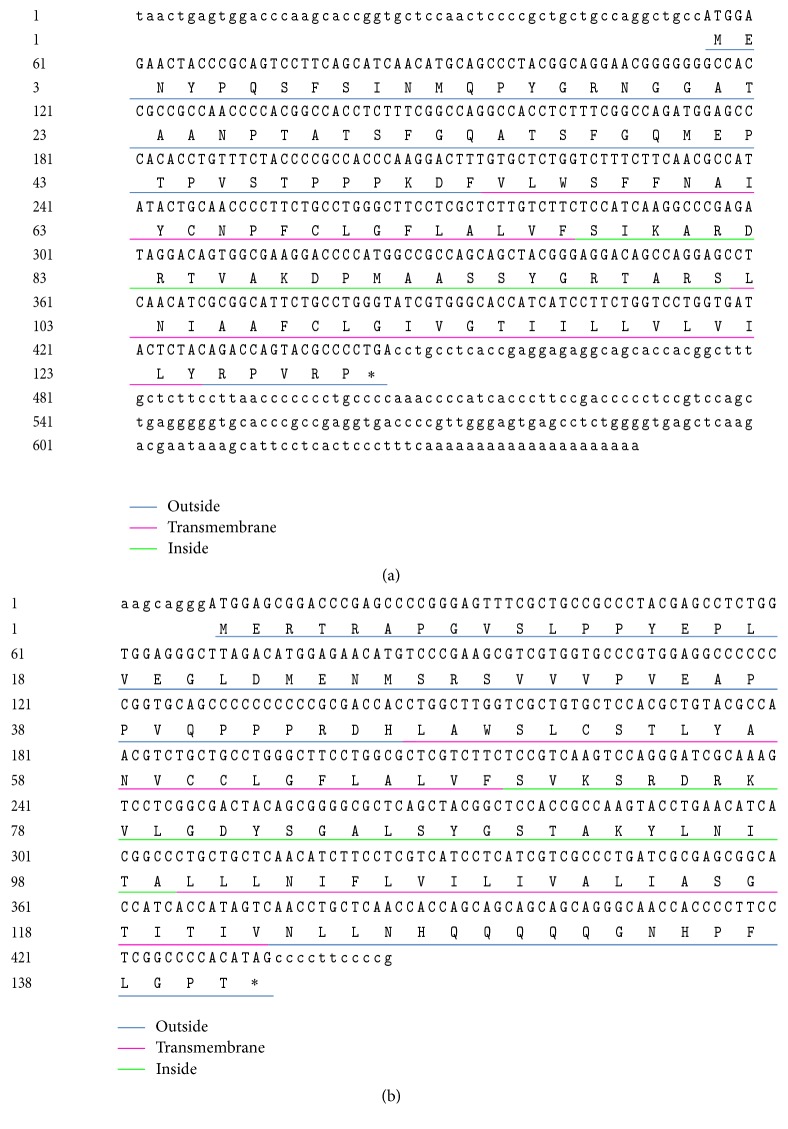
Nucleotide and amino acid sequences and putative topological structures of goose IFITM1 and IFITM3. The deduced amino acid sequences of goose IFITM1 (a) and IFITM3 (b) are presented below the nucleotide sequences. The ORFs are shown in uppercases letters, while the 5′- and 3′-UTR sequences are presented in lowercase letters. The stars represent the terminator codon. The five predicted protein domains of goose IFITM1 and IFITM3 are indicated by underlining. The blue, pink, and green lines represent extracellular domains, transmembrane helices, and intracellular domains, respectively.

**Figure 2:**
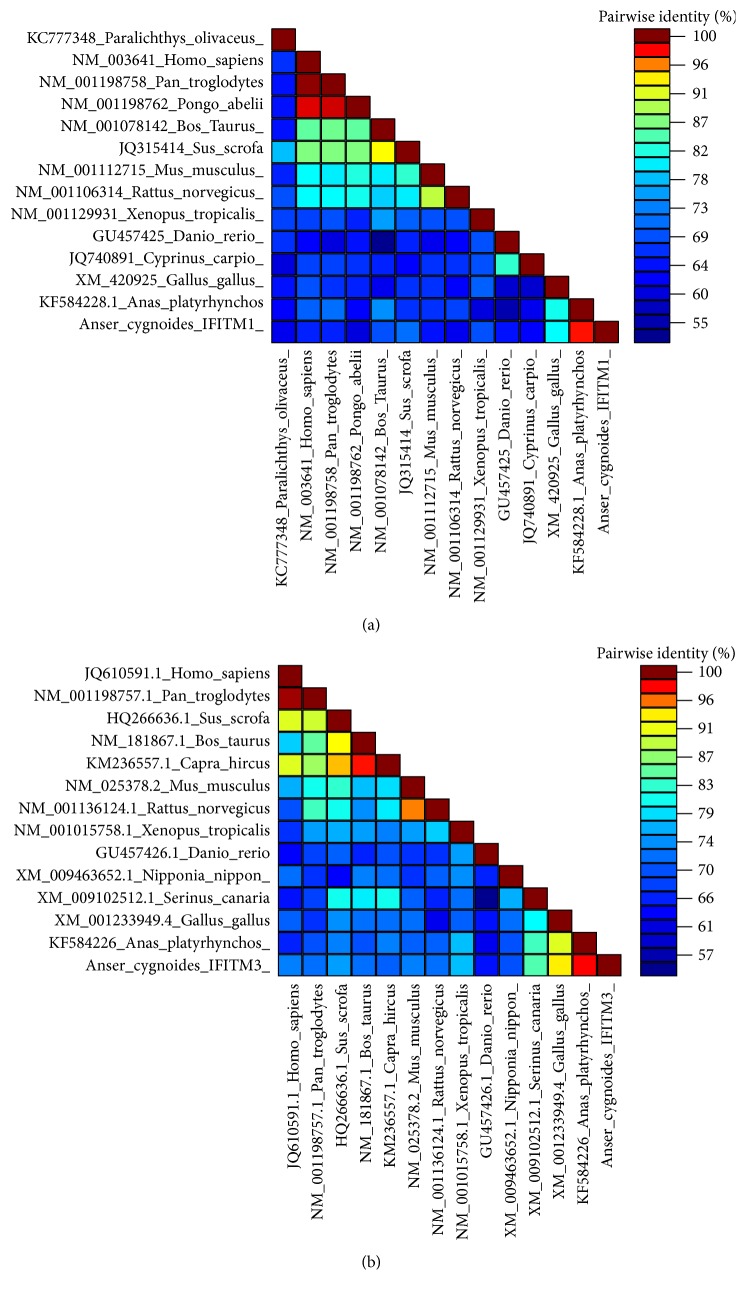
The 2D color-coded matrix of IFITM1 and IFITM3. The nucleotide sequences of IFITM1 (a) and IFITM3 (b) from various species were compared by pairwise alignment using the Species Demarcation Tool. The color spectrum scheme was assigned according to the pairwise identity scores. Goose IFITM1 and IFITM3 shared the highest identity with their respective counterparts in duck.

The full-length cDNA of goose IFITM1 (GenBank: KX594328) is 649 bp, with a 55-bp 5′-UTR, a 390-bp ORF, and a 204-bp 3′-UTR. The identified goose IFITM3 (GenBank: KX594327) contains a 426-bp CD and 19-bp UTRs (Figure 1). The CDs of goose IFITM1 and IFITM3 encode 129 and 141 amino acids (aa) with molecular weights of 14.0 kDa and 15.33 kDa, respectively. The nucleotide sequences from fourteen species were compared by pairwise sequence alignment analysis (Figure 2). The 2D color-coded matrix indicates that goose IFITM1 shares the highest identity with its counterpart in* Anas platyrhynchos* (GenBank: KF584228.1) (93.3%), followed by 70.6% shared identity with the* Gallus gallus* gene (GenBank: XM_420925), while it shared less than 60% identity with the sequences of primate genes. In addition, the nucleotide sequence of goose IFITM3 shared 94.5%, 83.4%, and 74.7% identity with the* Anas platyrhynchos*,* Gallus gallus*, and* Serinus canaria* counterparts, respectively. However, only 64.4% identity was shared between goose IFITM1 and IFITM3 (Tables S1 and S2 in Supplementary Material available online at https://doi.org/10.1155/2017/5149062). The pairwise identity scores of IFITM1 and IFITM3 are shown in Tables S1 and S2, respectively.

### 3.2. Characterization of IFITM1 and IFITM3 Proteins

**Figure 3:**
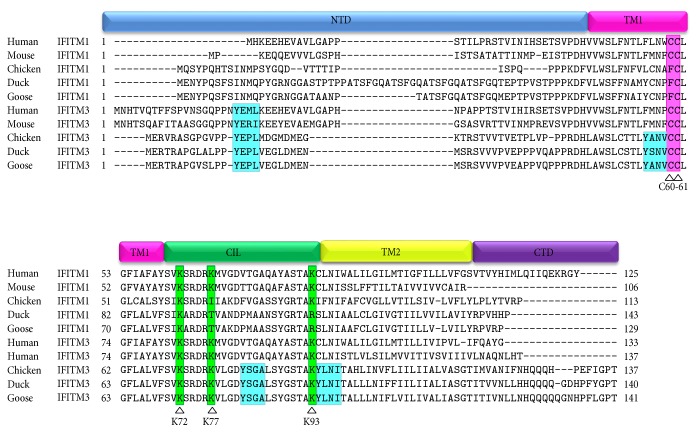
Multiple alignment analysis of IFITM1 and IFITM3 amino acid sequences. The amino acid sequences from goose, duck, chicken, mouse, and human were subjected to multiple alignment analysis using ClustalW. The proteins each consist of an N-terminal domain (NTD), transmembrane domain 1 (TM1), conserved intracellular loop (CIL), transmembrane domain 2 (TM2), and C-terminal domain (CTD). The blue highlights the YxxΦ motifs, which are absent in IFITM1. The two palmitoylated cysteines and three lysines serving as ubiquitination sites are indicated with pink and green, respectively.

Since these two molecules are transmembrane proteins, the topological structures were predicted by the TMHMM server. Two transmembrane helices were found in both proteins, which divided the IFITMs into five domains: a variable, hydrophobic N-terminal domain (NTD: IFITM1, 1–53 aa; IFITM3, 1–46 aa); a conserved hydrophobic transmembrane domain (TM1: IFITM1, 54–76 aa; IFITM3, 47–60 aa); a conserved intracellular loop (CIL: IFITM1, 77–101 aa; IFITM3, 70–99 aa); a variable, hydrophobic transmembrane domain (TMD2: IFITM1, 102–124 aa; IFITM3, 100–122 aa); and a short, highly variable C-terminal domain (CTD: IFITM1, 125–129 aa; IFITM3, 123–141 aa) (Figure 3). Neither the N- nor C-terminus of either goose IFITM1 or IFITM3 were predicted to be oriented toward the extracellular space. Multiple amino acid sequence alignments show several conserved residues that are critical for the antiviral function and localization of IFITMs. A highly conserved YxxΦ motif appears in the NTD of IFITM3, which promotes the internalization of IFITM3. However, IFITM1, with a comparatively short NTD, lacks this YxxΦ motif. In addition, three other YxxΦ motifs are conserved in IFITM3 of chickens, ducks, and geese. Two cysteines (C60 and C61) in TM1 are conserved in IFITM3 and undergo palmitoylation [31]. In addition, three lysines (K72, K77 and K93) that are the sites of ubiquitination are also present in IFITM3 [32] (Figure 3).

### 3.3. Phylogenetic Analysis of Goose IFITM1 and IFITM3

**Figure 4:**
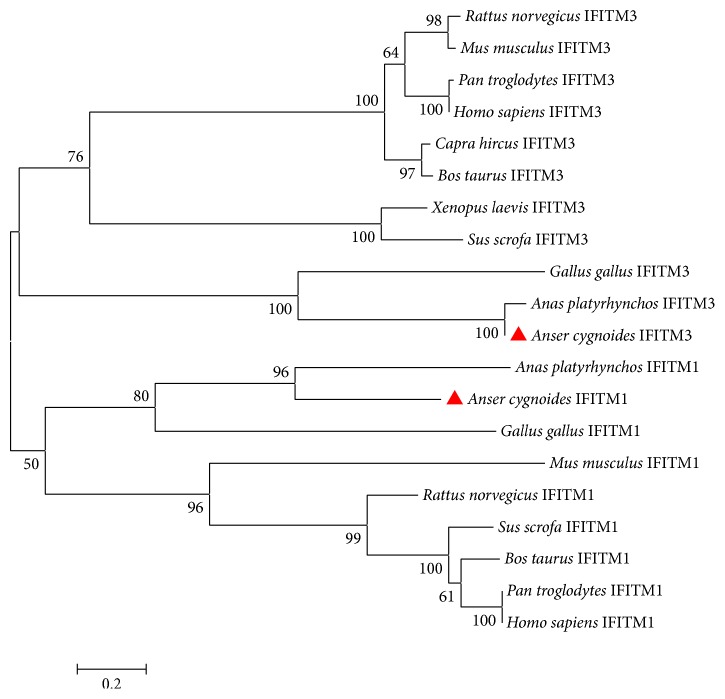
The phylogenetic evolutionary analysis of IFITM1 and IFITM3. The IFITM1 and IFITM3 clusters were divergent subgroups. The goose IFITMs, either IFITM1 or IFITM3, were located in the same monophyletic group with their respective counterparts in duck.

To explore the relationship of IFITM1 and IFITM3, as well as the evolutionary dynamics of goose IFITM1 and IFITM3, a phylogenetic tree was constructed using the amino acid sequences of IFITM1 and IFITM3 from diverse species. The phylogenetic tree indicated that IFITM1 and IFITM3 of diverse species were separated into two clusters. The avian IFITM1 and IFITM3 were clustered into two groups, respectively. Predictably, goose IFITM1 and IFITM3 were closely related to their homologs in ducks (Figure 4).

### 3.4. Tissue Distribution of Goose IFITM1 and IFITM3

**Figure 5:**
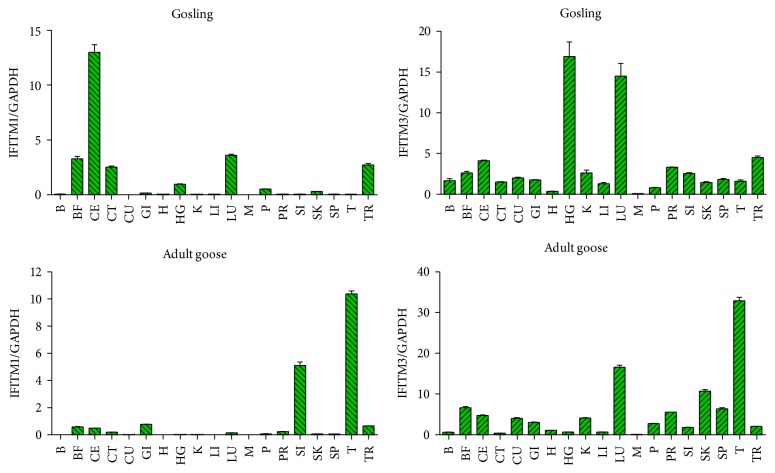
The tissue distribution profiles of goose IFITM1 and IFITM3. The constitutive expression levels of IFITM1 and IFITM3 in the brain (B), bursa of Fabricius (BF), cecum (CE), cecal tonsil (CT), gizzard (GI), heart (H), Harderian gland (HG), kidney (K), liver (LI), lung (LU), muscle (M), pancreas (P), proventriculus (PR), small intestine (SI), skin (SK), spleen (SP), thymus (T), and trachea (TR) were assessed by qRT-PCR.

The mRNA expression levels of IFITM1 and IFITM3 in eighteen tissues collected from healthy geese were assessed by qRT-PCR. In gosling, goose IFITM1 was highly expressed in the cecum, moderately expressed in the lung, bursa of Fabricius, trachea and cecal tonsil, and minimally expressed in the rest of the tissues. However, goose IFITM3 was widely expressed in all collected tissues. High relative expression levels of goose IFITM3 were detected in the Harderian gland and lung compared to the other tissues (Figure 5). Meanwhile, muscle tissue exhibited the lowest transcription level of goose IFITM3. In adult goose, comparatively high expression levels of goose IFITM1 were observed in the thymus and small intestine. In addition, the production of goose IFITM3 was easily detected in the thymus followed by the lung, skin, and bursa of Fabricius but was only faintly exhibited in the muscle tissue of adult goose (Figure 5).

### 3.5. The Expression Levels of Goose IFITM1 and IFITM3 in Challenged PBMCs

**Figure 6:**
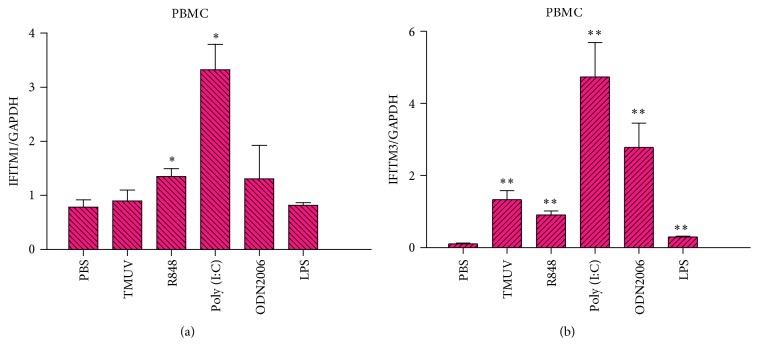
The effects of TMUV infection and TLRs agonist treatment on the expression of goose IFITMs in PBMCs. The transcription levels of goose IFITM1 and IFITM3 were quantified in triplicate in the goose PBMCs that were challenged with TMUV, R848, Poly (I:C), ODN2006, and LPS. The data are presented as the mean ± SEM (*n* = 3) and were analyzed with an unpaired two-tailed *t*-test: ^*∗*^*P* ≤ 0.05 and ^*∗∗*^*P* ≤ 0.01.

IFITMs have been reported to be broad-spectrum antiviral molecules [15, 33]. Once host cells are infected, the invasive pathogens are recognized by pattern-recognition receptors (PRRs) [34], which drive the production of IFNs. Subsequently, the interaction of IFNs and IFN receptors triggers the activation of ISGs such as IFITMs [1]. Here, whether goose IFITM1 and IFITM3 were activated in response to exogenous stimulation was monitored by qRT-PCR. As expected, goose IFITM1 and IFITM3 actively responded to treatment with either TMUV or TLRs agonists. TMUV and all the agonists led to extremely significant upregulation of goose IFITM3 in comparison with the PBS group (Figure 6(b)). However, only poly(I:C) and R848 effectively caused significant upregulation of goose IFITM1 (Figure 6(a)).

### 3.6. The Expression Profile of Goose IFITM1 and IFITM3 and Viral Copy Numbers in TMUV-Infected Goslings

**Figure 7:**
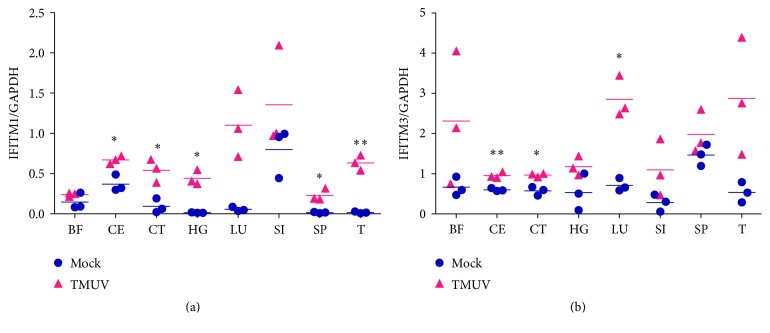
The effects of TMUV infection on the expression of goose IFITM1 and IFITM3 in vivo. Immune-related and epithelial tissues were collected, and the mRNA levels of goose IFITM1 and IFITM3 were quantified (normalized to goose GAPDH). Each dot represents an individual goose. The data are presented as the mean ± SEM (*n* = 3) and were analyzed with an unpaired two-tailed *t*-test: ^*∗*^*P* ≤ 0.05 and ^*∗∗*^*P* ≤ 0.01. Bursa of Fabricius: BF, cecum: CE, cecal tonsil: CT, Harderian gland: HG, lung: LU, small intestine: SI, spleen: SP, and thymus: T.

**Figure 8:**
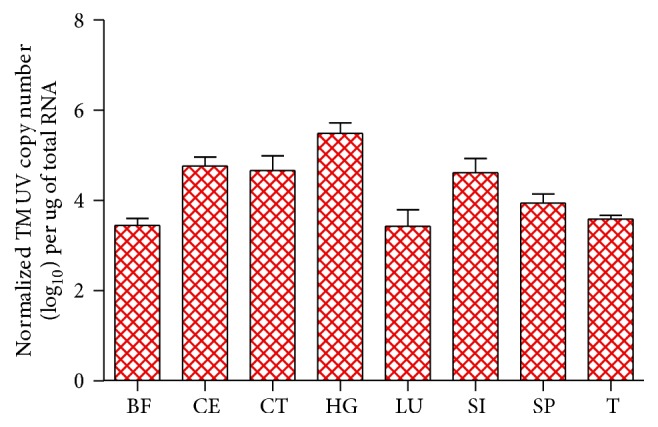
The detection of viral copy numbers in TMUV-infected goose tissues. The tissues mentioned in Figure 7 were analyzed for the corresponding viral copy numbers by an absolute quantification method in triplicate. The viral copy numbers (log_10_⁡ ) were normalized to 1 *μ*g of total RNA.

To investigate the impact of TMUV infection on the transcription levels of goose IFITM1 and IFITM3, the relative expression levels of immune-related and epithelial tissues from goslings treated with normal saline or infected with TMUV were examined. As shown in Figure 7, goose IFITM1 and IFITM3 were universally upregulated in response to TMUV infection. Compared to the mock-infected group, goose IFITM1 in the cecum, cecal tonsil, Harderian gland, and spleen was significantly upregulated in the TMUV-infected group. In addition, goose IFITM1 in the thymus was extremely significantly upregulated after TMUV infection (Figure 7(a)). Moreover, extremely significant upregulation of goose IFITM3 was observed in the cecum at 5 dpi with TMUV. Meanwhile, the transcription levels of goose IFITM3 were obviously upregulated in the cecal tonsils and lungs from TMUV-infected animals (Figure 7(b)). Furthermore, relatively high viral copy numbers were observed in these corresponding tissues, although the viral copy numbers in lung tissues were lower than those of other tissues (Figure 8).

## 4. Discussion

Since IFITMs, as cell-intrinsic restriction factors, were first identified to have a role in the restriction of influenza and flavivirus infection in 2009 [3], the antiviral function of IFITMs has been widely studied in various species, including human [35, 36], pig [37, 38], and mouse [39, 40]. However, in avian species, IFITMs were identified in only a small number of birds. The goose IFITM1 and IFITM3 identified here will facilitate a better understanding of the IFITM family in avian species.

As always, the nomenclature of IFITM1 and IFITM3 in chickens and ducks was confusing and controversial. According to the gene synteny with mammals, the predicted chicken IFITM3-like (GenBank: XM_420925) and chicken IFITM1-like (GenBank: XM_001233949) were renamed chicken IFITM1 and IFITM3, respectively. In addition, the names of the previously identified duck IFITM3 (GenBank: KF584228) and duck IFITM1 (GenBank: KF584226) were reversed. The pairwise sequence alignment analysis of nucleotide sequences showed that goose IFITM1 shares a much higher identity with its counterparts in duck (GenBank: KF584228) (93.3%) and chicken (GenBank: XM_420925) (70.6%) than with duck IFITM3 (GenBank: KF584226) (63.5%) and chicken IFTM3 (GenBank: XM_001233949) (59.4%). A similar result was observed in the nucleotide sequences alignment of goose IFITM3 with chicken IFITM1 and IFITM3, as well as duck IFITM1 and IFITM3 (Table S2). The phylogenetic analysis indicated that the IFITM1 and IFITM3 clusters were divergent subgroups. Goose IFITM1 and the renamed chicken IFITM1 and duck IFITM1, together with the mammalian IFITM1, were classified into the same subgroup. Meanwhile, goose IFITM3 and the renamed duck IFITM3 were located in the same monophyletic group, which was closely related to the renamed chicken IFITM3. In summary, the results revealed that it was necessary to rename the IFITM1 and IFITM3 in chicken and duck and that goose IFITM1 and IFITM3 have been conserved during evolution.

The minor IFITM3 allele (SNP rs12252-C), which is equivalent to the N-terminal 21-amino acid-truncated IFITM3, was strikingly enriched in patients who were severely infected with the 2009 pandemic H1N1 virus [35, 41]. In line with the above findings, VSV-G envelope protein-mediated virus entry could not be inhibited by the IFITM3 mutant (the N-terminal 21-amino acid deletion) [14]. These results highlight the important role of the N-terminal region of IFITM3 in antiviral functions [14]. In addition, the 20-YxxΦ-23 sorting signal in the N-terminal region is critical for the internalization of IFITM3 from the cell periphery to endosomal compartments where it acts to impede virus entry [42]. The N-terminal YxxΦ motif was observed in the N-terminal region of goose IFITM3. We predicted that the conserved motif in goose IFITM3 is also involved in the internalization and antiviral actions. Moreover, three other YxxΦ motifs that might be required for the endosomal localization and antiviral functions of duck IFITM3 [18] are also conserved in IFITM3 of chickens and geese. However, whether these YxxΦ motifs in goose IFITM3 are critical for correct cellular localization and inhibition of virus entry has yet to be confirmed.

Analysis of the tissue distribution of goose IFITM1 and IFITM3 in geese showed that both goose IFITM1 and IFITM3 were readily detected in some immune-related tissues, including the thymus, bursa of Fabricius, and Harderian gland. In addition, the expression of goose IFITM1 was restricted and primarily confined to the gastrointestinal tract tissues (cecum, small intestine, and gizzard), which was observed previously in both chickens [17] and ducks [18]. In contrast, goose IFITM3 was constitutively and ubiquitously expressed in various tissues. Notably, high expression levels of goose IFITM3 were observed in respiratory tract tissues (lung and trachea), the target tissues of infection with influenza A viruses, compared to the other tissues, which might contribute to the inhibition of influenza A virus replication [43]. It is notable that goose IFITM1 and IFITM3 both exhibit the lowest levels of expression in muscle tissues, similar to observations in chickens [17].

In vitro, we explored the immunological characteristics of goose IFITM1 and IFITM3. The mRNA level of IFITM3 in PBMCs sharply increased with TMUV infection and with R848, Poly (I:C), ODN 2006, or LPS treatment, although goose IFITM1 was prominently expressed only in the R848- and Poly (I:C)-stimulated groups. R848, Poly (I:C), ODN 2006, and LPS are known as TLR7, TLR3, TLR21, and TLR4 agonists, respectively. All four agonists and the TMUV genome can be recognized by PRRs, which then activate downstream signal transduction pathways triggering the production of cytokines, such as IFNs, that induce the transcription of ISGs, including IFITMs [1, 44–46].

Previous studies demonstrated that immune-related IFITMs were significantly upregulated in highly pathogenic avian influenza-infected ducks [18, 47]. However, the available information on whether IFITMs positively respond to TMUV infection is scant. Obviously, the expression levels of goose IFITM1 and IFITM3 displayed an upward trend in all the tested tissues, especially the cecum (Figure 7), where TMUV was primarily distributed (Figure 8). High viral copy numbers were also observed in the intestinal tract tissues of TMUV-infected ducks [48]. In addition, goose IFITM1 was intensively transcribed in the TMUV-infected spleen and thymus (Figure 7), where the significant upregulation of the IFNs was previously observed [48]. After TMUV infection, the virus was intensively located in the collected tissues, which reasonably explains why higher expression levels of goose IFITM1 and IFITM3 appeared in the TMUV-infected gosling tissues than those of the mock-infected group. In addition, the lowest viral copy numbers were seen in lung tissues, which might be attributed to the high expression level of IFITM3 in the TMUV-infected goose lungs. In light of the positive response of goose IFITM1 and IFITM3 to TMUV infection, we speculate that goose IFITM1 and IFITM3 might facilitate IFN-mediated defenses against TMUV.

## 5. Conclusion

Goose IFITM1 and IFITM3 were characterized for the first time. In addition, high mRNA levels of goose IFITM1 and IFITM3 were presented in some immune-related tissues. Meanwhile, goose IFITM1 and IFITM3 were primarily expressed in intestinal tract tissues and respiratory organs, respectively. Furthermore, goose IFITM1 and IFITM3 were activated in response to TMUV infection in vitro and in vivo. These results indicated that goose IFITM1 and IFITM3 might be potential antiviral effectors that restrict TMUV infection.

## Supplementary Material

Pairwise identity scores of Goose IFITM1 and IFITM3.

## Figures and Tables

**Table 1:** List of primers and sequences.

Primer name	Primer sequence (5′-3′)	Instruction
*IFITM1-F*	TCTA(C/G)CCCGCC(A/C)CCCAAG	Partial sequence
*IFITM1-R*	GGCTC(C/T)T(C/G)GCTGTCCTCC	
*IFITM3-F*	CCACCTGGCTTGGTCGCT	
*IFITM3-R*	GGCGATGATGAGGATGATG	

*AP*	CCAGTGAGCAGAGTGACGAGGACTCGAGCTCAAGC(T)18	3RACE
*AP1*	CCAGTGAGCAGAGTGACG	
*AP2*	GAGGACTCGAGCTCAAGC	

*AAP*	GGCCACGCGTCGACTACGGGIIGGGIIGGGIIGGGIIG	5RACE
*AUAP*	GGCCACGCGTCGACTAGTAC	

*IFITM1-5*′*GSP0*	TGGCTGTCCTCCCGT	5RACE-GSP
*IFITM1-5*′*GSP1*	GGTCCTTCGCCACTGTCCTATC	
*IFITM1-5*′*GSP2*	CGAGGAAGCCCAGGCAGAAG	
*IFITM3-5*′*GSP0*	GGCGATGATGAGGATG	
*IFITM3-5*′*GSP1*	GGAAGCCGAGGCAGCAGAC(A/G)TTG	
*IFITM3-5*′*GSP2*	GGAGCACAGCGACCAAGCCAG	

*IFITM1-3*′*GSP1*	GTGCTCTGGTCTTTCTTCAACG	3RACE-GSP
*IFITM1-3*′*GSP2*	CGAGATAGGACAGTGGCGAAC	

*IFITM1-5*′*F*	TGCTCCAACTCCCCGCTGCT	Complete coding sequence
*IFITM1-3*′*R*	CGTGGTGCTGCCTCTCCTC	
*IFITM3-5*′*F*	AAGCAGGGATGGAGCGGAC	
*IFITM3-3*′*R*	CGGGGAAGGGGCTA(T/A)GTG	

*IFITM1-qPCR-F*	TCTGGTCTTTCTTCAACGCC	Real-time PCR
*IFITM1-qPCR-R*	TTGAGGCTCCTGGCTGTCC	
*IFITM3-qPCR-F*	CCACCTGGCTTGGTCGCT	
*IFITM3-qPCR-R*	CGCTGTAGTCGCCGAGGA	
*GAPDH-qPCR-F*	CATCTTCCAGGAGCGCGACC	
*GAPDH-qPCR-R*	AGACACCGGTGGACTCCACA	
